# Mechanisms of Forgetting: A Systematic Review of Neurocognitive and Clinical Determinants

**DOI:** 10.3390/medicina62081550

**Published:** 2026-08-12

**Authors:** Mirza Masroor Ali Beg, Mohamed Aalilil, Awdhesh Kumar Mishra, Kwang-Hyun Baek, Ramachandran Vinayagam

**Affiliations:** 1 Faculty of Medicine, Ala-Too International University, Bishkek 720048, Kyrgyzstan; mirzamasroor.alibeg@alatoo.edu.kg (M.M.A.B.); alililmohamed60@gmail.com (M.A.); 2 Department of Biotechnology, Yeungnam University, 280 Daehak-Ro, Gyeongsan 38541, Gyeongbuk, Republic of Korea; awdhesh@ynu.ac.kr

**Keywords:** forgetting, intentional suppression, Alzheimer’s disease, Post-Traumatic Stress Disorder (PTSD), Major Depressive Disorder (MDD)

## Abstract

*Background and Objectives*: Forgetting has been a passive process of memory failure; an active and controlled neurocognitive process is critical for cognitive efficiency, goal-directed behaviors, and emotional coping. This systematic review aims to provide a human-based summary of the scientific understanding of forgetting from neurobiological and psychological perspectives. *Materials and Methods*: A literature review from 2000 – 2025 was performed using PubMed/MEDLINE, PsycINFO, and Scopus. Original peer-reviewed human-based studies that used behavioral tasks, neuroimaging methods, electroencephalography, and physiological stress inductions were considered. *Results*: Active control processes have been identified in the forgetting paradigm, and intentional forgetting involved prefrontal–hippocampal downregulation, whereas unintentional forgetting occurred due to limited resources, interference, and competition at the stage of information retrieval. Stress-induced disruption of the ability to recall memories operated specifically via executive and inhibitory functions. Pathological forgetting in mild cognitive impairment was explained by encoding impairments and poor consolidation, but not by accelerated long-term decay. Post-Traumatic Stress Disorder (PTSD) and Major Depressive Disorder (MDD) featured pathological forgetting associated with decreased specificity, negative bias, and poor prefrontal–medial temporal lobe inhibition, resulting in asymmetrical memory retention. Forgetting has been recognized as a dynamic and multidetermined process, which involves executive control, hippocampal–cortical interactions, and emotional modulation. Different pathologies of forgetting occur depending on the disrupted domain, indicating that differential forgetting patterns specific to particular domains, such as encoding failures in Mild Cognitive Impairment (MCI) and inhibitory deficits in PTSD, may have implications for differential diagnoses and interventions. *Conclusions*: Executive and emotion-regulatory dysfunction leads to maladaptive forgetting and is characterized by less specificity, more interference, and weaker inhibition. Disrupted hippocampal amygdalar prefrontal networks, in disorders like PTSD and MDD, disrupt memory suppression, resulting in intrusive emotional memories with loss of context.

## 1. Introduction

**Figure 1:**
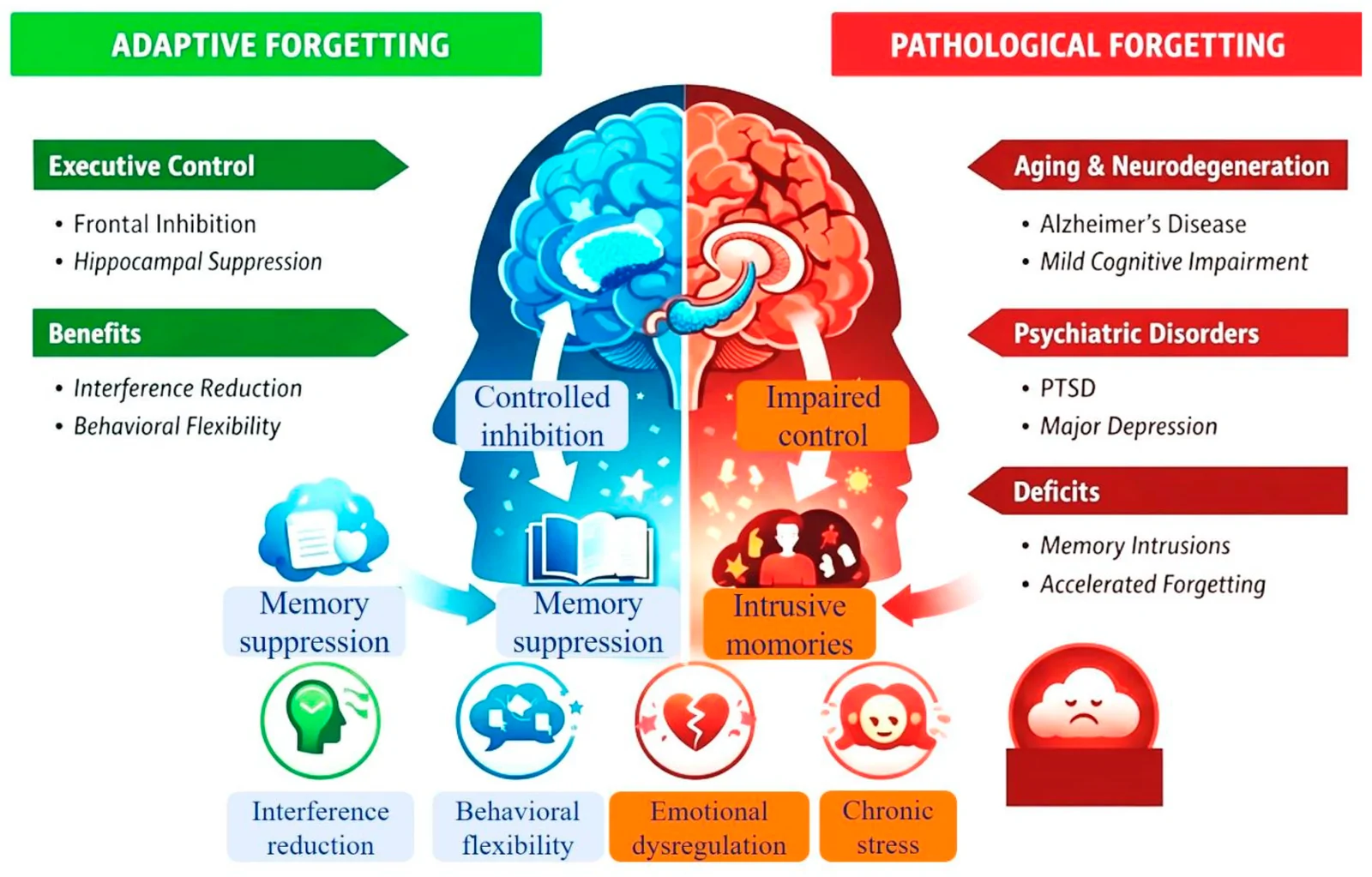
Original schematic created by the authors: conceptual framework of human forgetting across cognitive, emotional, and clinical domains.

Memory is a basic cognitive process that allows encoding, storage, and retrieval of information, and study showed forgetting is a vital and adaptive pattern of memory processes, not a failure to remember. Experimental psychology and neuroscience evidence show that forgetting can be controlled to maximize cognitive efficiency, minimize interference, and achieve goal-directed behavior (Figure 1) [1,2].

Historically, theories on forgetting were categorized into three classical categories. In decay theory, forgetting was defined as weakening of memory traces due to lack of rehearsing without any interference from any other cognitive processes. In interference theory, forgetting occurred when memories interfered with each other while being encoded or retrieved [3].

According to “retrieval failure theory,” information that is lost in terms of memory usually exists in the memory, but for a temporary or permanent time period it cannot be retrieved due to inconsistency between cues used during the encoding process and cues used during the retrieval process [4].

Memory suppression has been extensively studied using directed forgetting paradigms, employing the item method and list method, which reveal that people can selectively prevent the encoding or retrieval of information [5,6]. Electroencephalography (EEG) and event-related potentials (ERP) studies have revealed that forgetting is linked to specific brain activity patterns, especially linked to frontal control mechanisms [7,8]. The study suggested that top-down executive control is essential in controlling memory processes. Moreover, studies pinpointing a hotspot for encoding verbal memory in the left anterior prefrontal cortex underscore the area’s role in regulating memory formation and suppression [9]. Patients with major depressive disorder have modified neural associations when interacting with directed forgetting exercises, especially in processing emotional information that may be associated with the development of enduring negative cognitive biases [10]. In the same manner, those with very high superior autobiographical memory also demonstrate variation in brain activity during active forgetting, indicating difficulty in suppressing highly detailed personal memories [11]. The dynamics of cognitive load and working memory are also vital in the development of forgetting. Prospective memory performance is influenced by increased cognitive demands, resulting in higher commission error rates when people are unable to suppress no-longer-relevant intentions [12]. Cognitive load and task characteristics, including focus, also interact with one another and impact prospective memory outcomes [4]. Furthermore, recent studies indicate that the rate of forgetting in working memory is adaptively controlled according to task demands, which is evidence of a flexible system to balance retention and elimination of information [4]. Other processes that differ with age include forgetting processes, which differ between the lifespan and in pathological situations. Aging research suggests that a faster rate of long-term forgetting can be one of the first signs of deterioration in cognitive functions, even in otherwise healthy participants [13]. Likewise, the progression of Alzheimer’s disease is marked by strong forgetting, which may not be accounted for by retrieval impairment solely, indicating that memory consolidation and memory storage are impaired [14]. Besides cognitive and clinical aspects, studies have examined the interference and neural efficiency in forgetting. It has been indicated that forgetting in visual long-term memory can be caused by decay and interference, whereby information competing with that in memory interferes with memory retention [3]. Neural fatigue is another factor that has been proposed to affect memory encoding, as declining hippocampal performance results in worse memory encoding over time [15]. Moreover, studies on fear memory have shown that the brain dynamically balances competing memory systems with time, and thus enables adaptive responses to evolving environments [16]. The evidence presented by behavioral, neurophysiological, and clinical studies together shows that forgetting is a very dynamic and controlled process. It is affected by the deliberate control processes, emotional and cognitive factors, and neurological factors. Through selective attenuation or suppression of irrelevant information, forgetting allows people to be cognitively efficient and adapt to complex environments. Therefore, the present systematic review seeks to synthesize the human evidence on behavioral paradigms, neuroimaging, electrophysiology, and clinical research findings to clarify the neurobiological, functional, and clinical aspects of forgetting. This review offers a basis for better diagnostic assessment, disease progression, and pathological and neurological population-informed therapeutic choices by differentiating adaptive and pathological forgetting. For instance, differentiating between encoding deficits that characterize early-stage mild cognitive impairment and inhibition deficits that characterize PTSD could help in determining whether to opt for memory training strategies or inhibition control-targeted strategies like prefrontal neurostimulation. The primary objective of this review article was to determine whether forgetting in humans represents a passive decay of memory traces or an active, regulated neurocognitive process, and to examine how this process is altered across healthy, aging, and clinical populations.

## 2. Materials and Methods

### 2.1. Study Design and Study Selection

**Figure 2:**
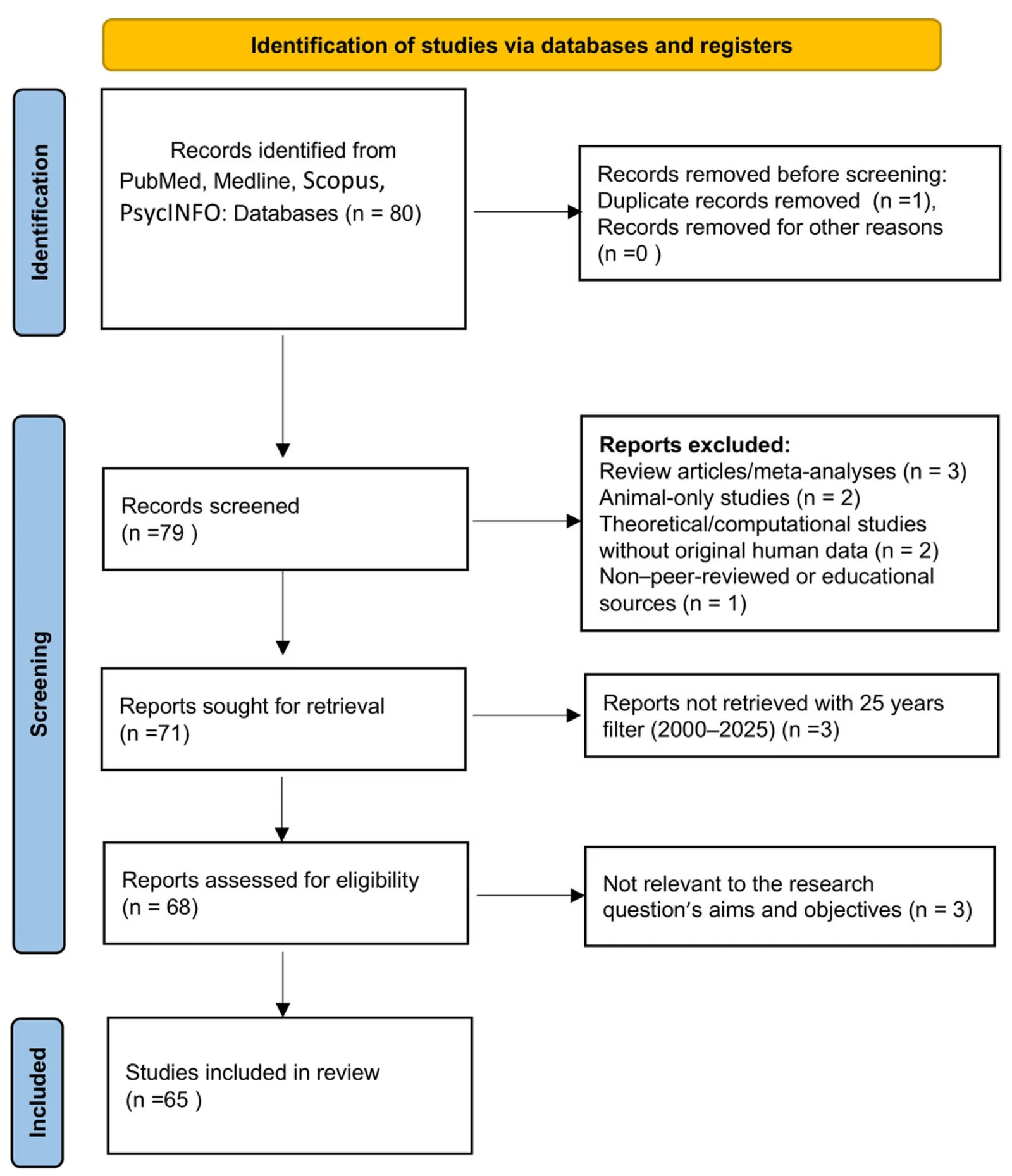
The PRISMA flow diagram showing the identification, screening, and study selection process for forgetting and neurocognitive.

The present systematic review was performed in accordance with Preferred Reporting Items for Systematic Reviews and Meta-Analyses (PRISMA) 2020 guidelines. The objective was to synthesize human evidence on the neurobiological and psychological mechanisms of forgetting across healthy, aging and clinical populations. A total of systematic database searches resulted in 80 records on PubMed/MEDLINE, Scopus, and PsycINFO. Following the deletion of a single duplicate, 79 records were screened with regard to titles and abstracts. Eight of them were eliminated according to predefined criteria (review or meta-analysis, *n* = 3; animal-only, *n* = 2; theoretical or non-empirical human, *n* = 2; non-peer-reviewed, *n* = 1). Then, 68 full-text articles were evaluated based on their eligibility. Three articles were not available within the predetermined publication range (2000 – 2025), and three more articles were filtered out as not fitting the objectives of the review. Therefore, 65 studies were included in the final qualitative synthesis and met all the requirements of inclusion, as described in the PRISMA flow diagram (Figure 2, Supplementary Materials File S1).

### 2.2. Search Strategy

The extensive literature search was performed in PubMed/MEDLINE, PsycINFO, and Scopus to find the related studies published between January 2000 and December 2025.

The search strategy involved “forgetting mechanisms OR adaptive forgetting, memory suppression OR directed forgetting, retrieval-induced forgetting, hippocampus, prefrontal cortex, memory, stress, memory retrieval, pathological forgetting, sleep and memory consolidation, Boolean operators were used to combine and refine search terms. Furthermore, reference lists of all the articles included were manually filtered to detect any additional studies that could be considered relevant and may have been overlooked during the first database search.

### 2.3. Criteria of Selection and Data Extraction

As per predefined criteria, studies included peer-reviewed original research involving human participants and focused on mechanisms of forgetting or remembering. Eligible studies employed established methodologies such as behavioral paradigms, cognitive testing, neuroimaging techniques (including fMRI and PET), electrophysiological approaches (such as EEG/ERP or intracranial EEG), or physiological stress manipulation. Additionally, studies were required to provide mechanistic, psychological, or clinical interpretations of memory processes and to be published in English between 2000 and 2025. Studies such as reviews, meta-analyses, editorials, commentaries, and dissertations; studies that were done in animals; descriptive memory studies that were not interpreted mechanistically; articles that were not peer-reviewed or educational, and were not published in English were excluded. The data extraction was carried out according to the PRISMA 2020 protocol. The variables for extraction were study design, participant characteristics, cognitive/neurobiological paradigm used, experimental condition, outcome measure, suggested mechanism of forgetting, and possible clinical or functional consequences of the forgetting process. Validated scales were used to assess the quality of the methodology and risk of bias in the selected studies depending on their design according to the PRISMA guidelines. Methodological quality check was performed by using Cochrane Risk of Bias 2.0 for randomized controlled trials and the Newcastle–Ottawa Scale (NOS) for observational studies. Randomized controlled trials were evaluated using the Cochrane Risk of Bias 2.0 tool and observational studies were conducted using the Newcastle–Ottawa Scale (NOS).

## 3. Results

In selected human studies, forgetting was defined as a multidimensional cognitive process rather than a consistent loss of information. The two main dimensions in which forgetting has always been classified according to experimental paradigms are [1] intentionality, i.e., intentional and unintentional forgetting, and [2] the underlying cognitive mechanism in which it occurs, i.e., retrieval competition, attentional or cognitive resource limitations, and inhibitory control processes [1,2,5,6,7].

### 3.1. Operational Definitions of Forgetting

**Figure 3:**
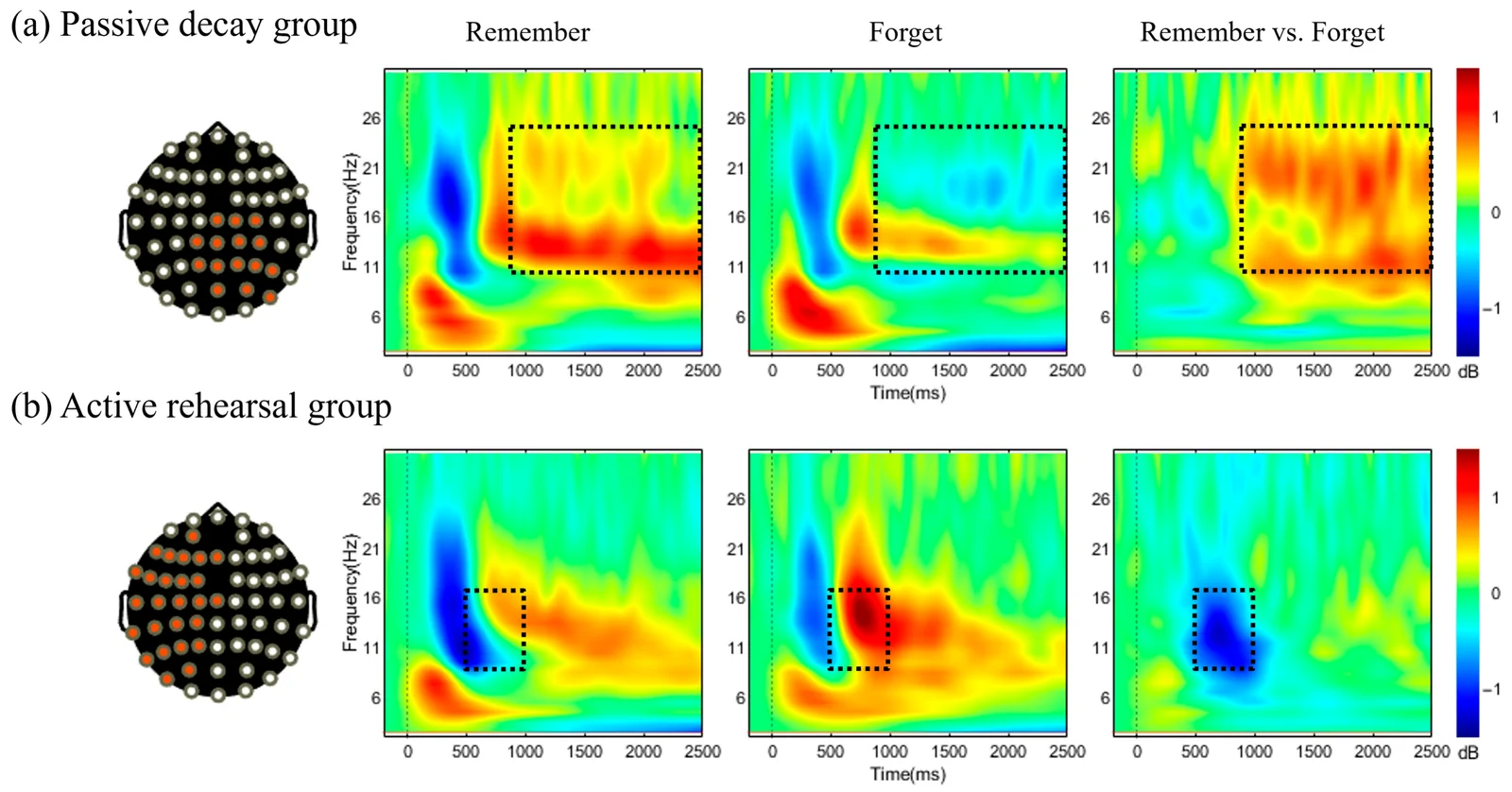
Time–frequency results for the study phase. The channel clusters appeared as red dots on the scalp plot, and the average of these channels was used to generate the time–frequency plot. The dotted lines indicate the moment of cue presentation, and the dotted boxes denote significant clusters. (**a**) The passive decay group identified a cluster over right posterior regions between 870 and 2500 ms, spanning approximately 9–25 Hz. (**b**) The active rehearsal group identified a cluster over left hemisphere regions between 500 and 1000 ms, spanning approximately 8–16 Hz [8]. The Authors. Distributed under the Creative Commons Attribution 4.0 License (CC BY 4.0).

Forgetting was defined and operationalized across multiple paradigms in three main ways. First, performance-based definitions, exemplified by Pan and Li [8], defined forgetting as a measurable decrease in delayed recall for items cued with a forgetting instruction relative to items cued to be remembered, demonstrating that forgetting instructions can be used to change accessibility (Figure 3). Second, accessibility definitions, as illustrated in the work of De Jong et al. [4], demonstrated that forgetting can occur in the absence of encoding failure, as a loss of accessibility at longer retention intervals or under high interference conditions, consistent with a model in which forgetting is due to retrieval failure. Third, contrast definitions, used in neuroimaging studies such as Santangelo et al. [11], defined forgetting by contrasting brain activity during successful and unsuccessful memory retrieval, showing changes in prefrontal and hippocampal activity during suppression or interference. Thus, across paradigms, forgetting was defined as a change in accessibility, influenced by control processes and task demands.

### 3.2. Intentional Versus Unintentional Forgetting

The two broad categories of forgetting are intentional and unintentional. Intentional forgetting is deliberate suppression of information, and studies using the directed-forgetting paradigm and think/no-think paradigm, respectively, have consistently reported decreased recall for to-be-forgotten items, increased activation of frontal control regions, and decreased hippocampal reactivation during suppression. Santangelo et al. [11] replicated these findings, showing reduced recall for items cued for suppression, compared to baseline. Unintentional forgetting, however, occurs under conditions where attention is divided, working memory is loaded, interference occurs or task demands shift. Cortese et al. [16] demonstrated that changing task demands resulted in participants decreasing their reliance on previously encoded information, reflecting unintentional forgetting due to dynamic task demands. De Jong et al. [4] also demonstrated unintentional forgetting in the context of rising working-memory demands, despite participants’ ability to intentionally control memory under low loads.

From a behavioral standpoint, the individuals performing in directed forgetting experiments demonstrated a highly significant difference in accuracy of recall between to-be-forgotten and to-be-remembered items, as well as higher speed of responses and decreased intrusion errors in conditions of intentional suppression [5,6]. Thus, the behavioral evidence proves that forgetting is systematic and controlled [1].

### 3.3. Process-Specific Mechanisms of Forgetting

Retrieval competition is important, in which intentional recall of some information strengthens the recall of that information while decreasing recall of related information [4,8,16]. Another mechanism is inhibitory control, where deliberate suppression of memories activates prefrontal areas and deactivates the hippocampus, impairing recall [10]. Resource limitations are also important for forgetting, as insufficient attention or processing resources are available under conditions of high task demands or changing context [4,8]. Forgetting also arises from context-dependent retrieval impairment, where discrepancies between encoding and retrieval contexts [particularly in emotional situations] impair recall [10]. Lastly, in older adults and patients, forgetting is often associated with inefficient encoding, with less consistent initial encoding, rather than increased memory decay, contributing to poorer memory performance [16]. From the above studies, the mechanisms of forgetting appeared to be heterogeneous and task-dependent, as several studies revealed results that are compatible with cognitive control of forgetting, where the mechanism involved inhibition, attention, interference, and contextual factors [4,8,16]. These results are not an established opinion; however, there are other theories for forgetting, including the theories based on decay, retrieval failure, and system consolidation of memory.

### 3.4. Anatomy of Memory and Forgetting in Human Studies

#### 3.4.1. Brain Regions Involved in Human Memory

Brain imaging and direct electrophysiological recordings have established that successful memory encoding and forgetting rely on interactions between prefrontal, medial temporal, and limbic regions. In a large intracranial EEG study of 135 epilepsy patients, a focal subsequent memory effect was observed in the left anterior prefrontal cortex, at the border between Broca area and the frontal pole, where power in the low-theta frequency band (3–5 Hz) consistently discriminated between subsequently remembered and forgotten words [9]. This site had higher predictive accuracy than other brain regions and was therefore identified as a key site for successful encoding. Supporting fMRI findings have identified the role of the medial temporal lobe (MTL). In trauma-exposed adults, patients with PTSD exhibited lower left hippocampal and amygdala activation during the encoding of emotional images, higher rates of false alarms for new items, and lower left hippocampal activity was associated with PTSD severity, suggesting that encoding difficulties in stress-related disorders reflect disrupted MTL activity [17]. In healthy adults, studies of emotional memory formation have demonstrated a functional distinction between MTL subregions: amygdala activity predicted memory of emotional items, the entorhinal cortex supported item memory, and the hippocampus supported contextual memory [18], explaining why emotional information might be retained while contextual information is lost. Multivariate fMRI has also demonstrated distributed cortical contributions, where category-specific patterns (faces vs. scenes) in temporal and prefrontal cortices during encoding predicted subsequent memory accuracy, suggesting that memory encoding is dependent on the strength of representational patterns rather than on localized activation [19].

#### 3.4.2. Neural Mechanisms of Memory Encoding

Memory encoding is a dynamic process that changes across trials and depending on prior encoding success. Intracranial EEG research has shown region-specific characteristics of encoding: while successful prior encoding resulted in decreased high-frequency activity (HFA) in the hippocampus, the opposite pattern was found in the dorsolateral prefrontal cortex (DLPFC) [15]. This suggests that the hippocampus and the prefrontal cortex play different roles in encoding. In line with these findings, functional near-infrared spectroscopy (fNIRS) research showed stage-specific activity of the prefrontal cortex: ventrolateral PFC (VLPFC) showed increasing activity throughout encoding, whereas retrieval involved a more extensive prefrontal network including DLPFC and frontopolar cortex [20].

#### 3.4.3. Neural Correlates of Memory Storage and Consolidation

Memory consolidation involves encoding-dependent hippocampal activation and offline reactivation during sleep. A directed forgetting fMRI study demonstrated that items to be remembered were preferentially consolidated during sleep, with encoding-related hippocampal activity predicting items that benefit from sleep consolidation [21]. Further electrophysiological evidence from intracranial EEG recordings also showed stimulus-specific replay in hippocampal ripples in non-REM (nREM) sleep. Importantly, late-encoding gamma patterns were replayed for items that were later remembered, but early encoding replay was boosted for items that were later forgotten [22].

#### 3.4.4. Neural Correlates of Retrieval and Retrieval Failure

Successful retrieval is enabled by large-scale connectivity between the medial prefrontal cortex (mPFC), the medial temporal lobe (MTL), and frontoparietal networks. An fMRI study with dynamic causal modeling found that autobiographical memory retrieval involved four interconnected networks: an mPFC network involved in self-referential processing, an MTL network involved in memory construction, a frontoparietal network involved in episodic search, and a cingulo-opercular network involved in goal maintenance, with the mPFC serving as a driver that alters connectivity depending on retrieval success [23]. Inability to retrieve memories is often related to inhibitory control, as Penolazzi et al. demonstrated that cathodal TMS on the right DLPFC completely removed retrieval-induced forgetting, validating that prefrontal inhibitory control is crucial for suppressing competing traces [24]. Further, it was observed that mPFC tDCS led to increased retrieval of competing memories and decreased retrieval-induced forgetting, which was associated with early beta-desynchronization in the left DLPFC and subsequently the parietal cortex, oscillatory patterns associated with successful retrieval [25].

### 3.5. Forgetting and Retrieval Failures Under Stress

Across the studies reviewed, stress was found to be a selective modulator of memory retrieval, affecting recall but not encoding. These findings were replicated in both nonclinical and clinical populations and were mediated by decreases in retrieval accessibility, increases in cognitive load, and decreases in inhibitory control.

#### 3.5.1. Memory Impacts of Acute and Chronic Stress

Acute stress (caused by emotional events, time pressure, and performance pressure) was generally linked to lower recall accuracy, even when encoding was unaffected. For example, Chen et al. [10] showed that emotionally stressful contexts selectively affect retrieval relative to neutral contexts. Likewise, task-specific stressors administered just before or during retrieval increased errors and lowered recall accuracy [3,26], suggesting that forgetting under stress is mostly due to retrieval, rather than encoding, impairments. Long-term stress, while less often investigated, demonstrated ongoing decreases in recall stability over multiple testing sessions. This was attributed to attentional and executive processing rather than cognitive impairment [27,28].

#### 3.5.2. Stress-Induced Retrieval-Specific Deficits

Other experiments that manipulate the timing of stress also support the direct effect of stress on retrieval. It has been observed that stress increases cognitive load and decreases reliance on previously stored information [16], while stress-related working memory load interferes with retrieval, but not encoding, of stored memories [4]. Memory under stress is usually forgotten in ways such as delayed response times, reduced retrieval accuracy, and greater interference from distractors. In addition, there are effects of emotional modulation, with fear- and anger-related cues disrupting inhibitory control more than neutral cues [29].

#### 3.5.3. Cortisol and Stress-Induced Forgetting

**Figure 4:**
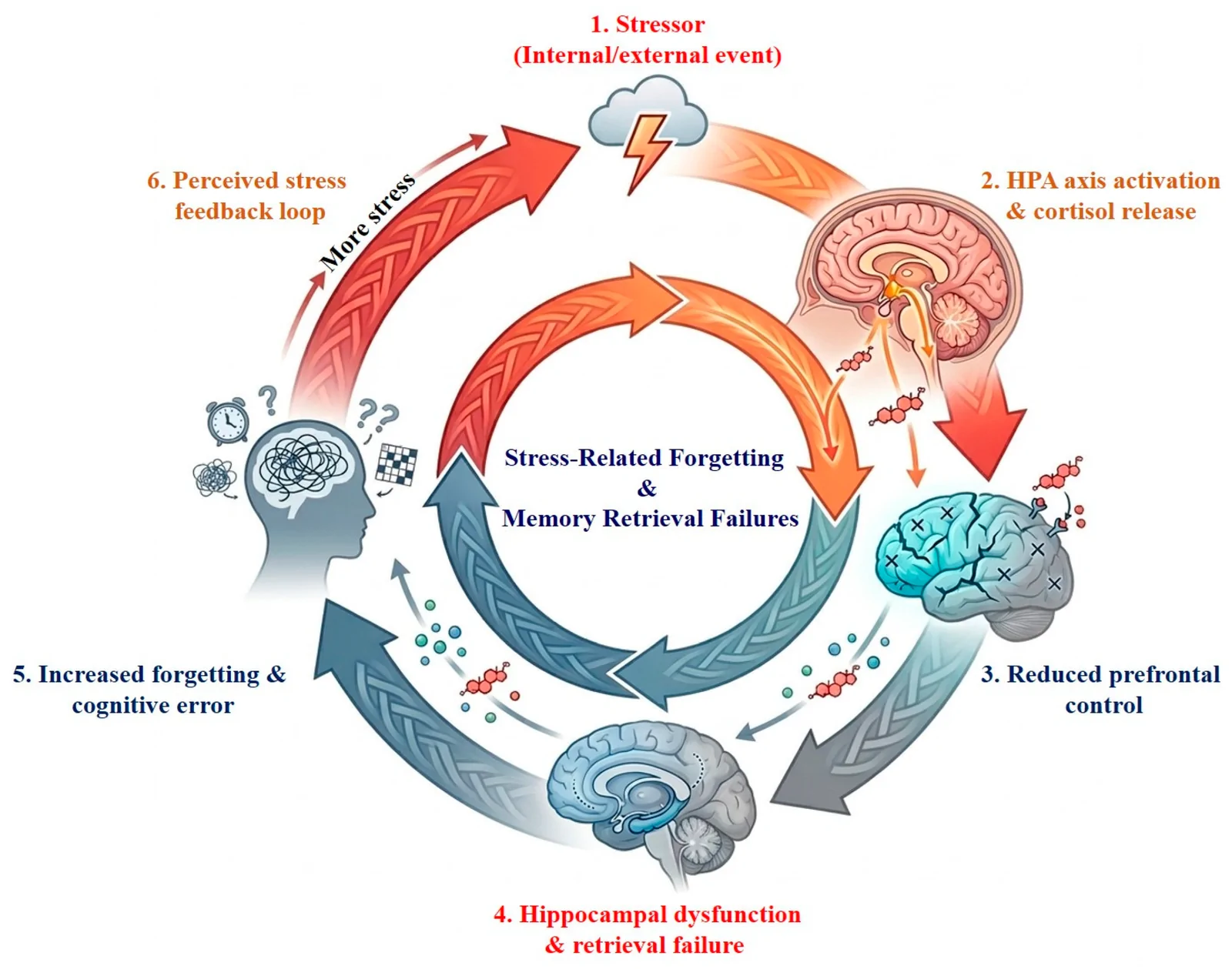
Original schematic created by the authors, illustrating the vicious cycle of stress and forgetfulness, presenting interplay between stress hormones, prefrontal control, and hippocampal dysfunction. High-Frequency Activity (HFA).

The physiological effects of stress, including effects of cortisol, also imply a retrieval impairment. While not always directly assessed, responses under stress reflect hippocampal-dependent forgetting. Stressed participants display impaired recall accuracy, with relatively intact recognition and immediate recall [26,27,30], which is in line with the effects of glucocorticoids on different memory systems. Crucially, forgetting through inhibition is not eliminated by stress, and retrieval-induced forgetting can still be observed [31]. Moreover, stress effects may differ between men and women, with cortisol responders being more sensitive to recall performance [32]. Stress-induced forgetting can be explained as a context-specific failure to recall due to disruptions in executive processes, greater sensitivity to interference, and availability of attentional resources rather than permanent loss or decay of the memory (Figure 4).

### 3.6. Cognitive Load, Attention, and Prospective Memory Failures

**Figure 5:**
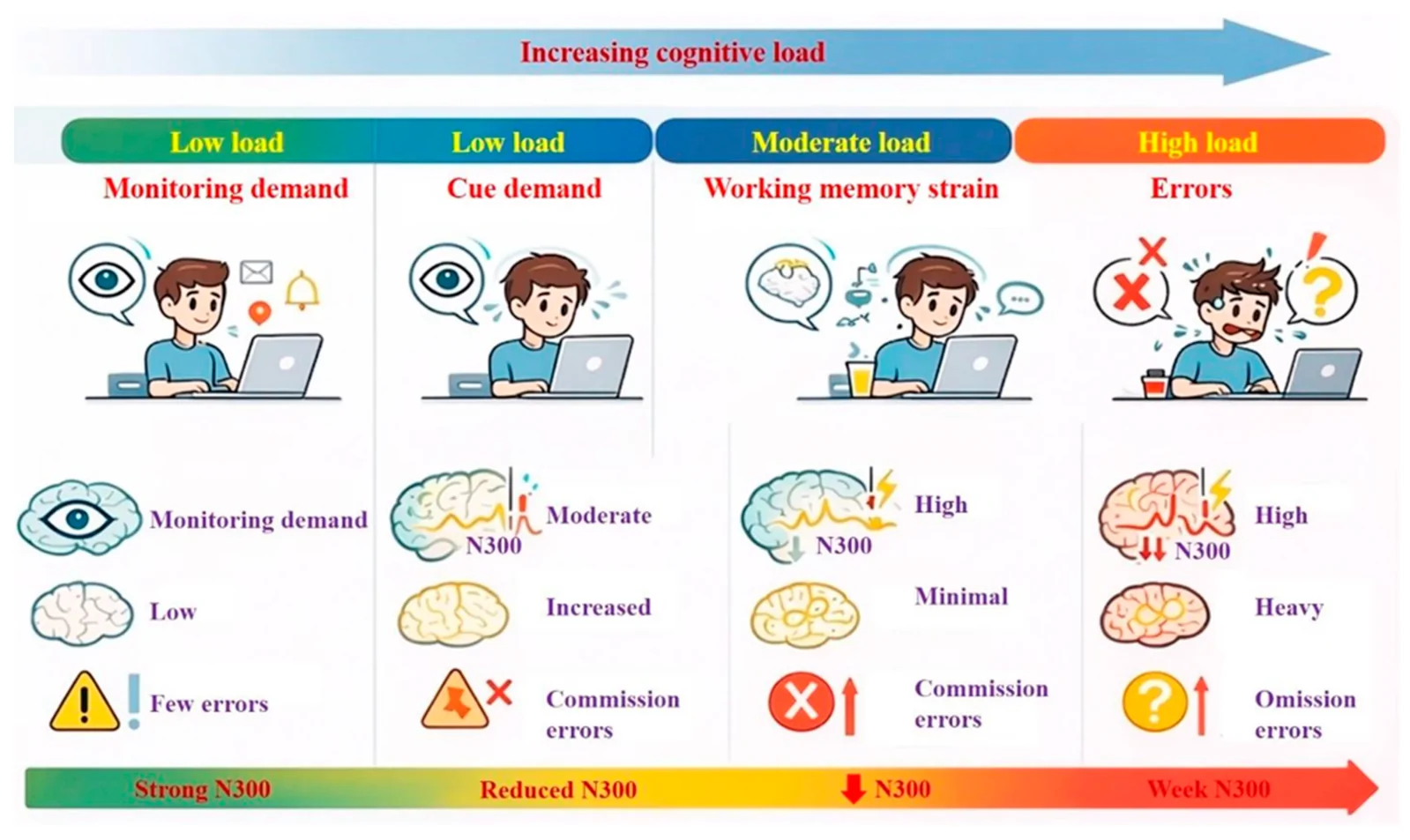
Original schematic created by the authors demonstrated the connection between increasing cognitive load and prospective memory failure markers.

Human prospective memory and working memory are highly resource-dependent processes. Across the studies reviewed, forgetting appeared as a systematic consequence of increased cognitive load, attentional competition, and interference, rather than being a random event. The results suggest that forgetting is primarily a consequence of the limitations in executive control, particularly those necessary for the maintenance, monitoring, and execution of deferred intentions (Figure 5).

#### 3.6.1. Prospective Memory Commission/Omission Errors Under Cognitive Load

Cognitive load profoundly affects prospective memory, with increased rates of commission and omission errors. There is evidence from Matos et al. [12] that shows a non-linear effect, with commission errors (inappropriate enactment of time-based intentions) at their highest under moderate load (74%) than under no load (40%) and remaining stable under high load. This suggests that errors are caused not only by monitoring failure but by lingering activation of intentions, consistent with a spontaneous retrieval rather than a control-based mechanism. In line with this, prospective memory performance is impaired under high cognitive load, especially under non-focal conditions, where monitoring demands outpace working memory capacity [33]. Khan et al. [34] also demonstrated that self-initiated time-based prospective memory, which is less supported by external cues, is more impaired by increased load, whereas prospective memory for events that are cued by external events is spared.

#### 3.6.2. Differentiated Load Components and Attentional Coordination in Prospective Memory

Based on their study, Meier and Zimmermann elaborated upon dividing the cognitive load into three different types: prospective load (PL), retrospective load (RL), and ongoing task load (OTL). They found that PL mostly heightened the need for monitoring and, as a consequence, reduced the speed of performance on the ongoing task. On the other hand, both RL and OTL only affected the retrieval of the retrospective part of the intention, that is, which action to perform. This three-way distinction suggests that forgetting in prospective memory tasks is caused by a number of load-sensitive bottlenecks rather than by one single failure point, thus pointing out the importance of componential models of memory failure [35]. Research conducted by Rummel et al. also demonstrated that when the prospective memory response overlapped with the ongoing task response, prospective memory performance was much higher than if there was no overlap; however, if there was no overlap between the prospective memory response and the ongoing task response, the correlation between working memory capacity and prospective memory responses was significant. As such, the results demonstrate that the stage at which failure occurs most often in relation to completing the intended action (i.e., coordinating the intended action with the ongoing task) requires some degree of attentional flexibility [36].

#### 3.6.3. Neural Correlates and Vulnerable Working Memory in the Context of Load and Stress

There is electrophysiological evidence that suggests that prospective memory is influenced by working memory load. It has been demonstrated that the effects of working memory load on the N300 component of event-related potentials (ERPs), which is a marker of cue detection, and that higher levels of working memory load resulted in poorer prospective memory performance [37]. The sustained neural modulation produced by the prospective memory load suggests that resources are continuously used to maintain one’s intention. Working memory is particularly susceptible to high levels of stress and load. Psychosocial stress specifically impaired working memory performance under high loads; however, low-load conditions produced stable working memory performance, with cortisol levels correlating with the impairment under high-load conditions [38]. Consistent with that, Lupien et al. [39] also extended the finding that hydrocortisone administration, in other words, the administration of a glucocorticoid, produced an impairment of working memory but not of the ability to sustain attention (vigilance) or of the ability to recall or recognize information (declarative memory). Again, this provides evidence of the susceptibility of working memory performance to load-specific challenges. Altogether, these data confirm that the limitations of attention, working memory functions, and stress-induced neuroendocrine responses create neural bottlenecks that undoubtedly contribute to forgetting [39].

#### 3.6.4. Load-Dependent Interference, Adaptive Forgetting, and Clinical Vulnerabilities

Cognitive load influences interference-related forgetting, according to Altmann and Gray [40]. The pattern of delay indicated that the mechanisms of adaptive decay, which serve to decrease the interference of outdated information, were in effect. Similarly, Sun et al. [41] found that the similarity of the distracting material determined the type of forgetting: highly dissimilar interference caused erasure of memory [higher levels of guessing], while moderately similar interference resulted in a loss of precision. Therefore, interfering does not merely diminish a memory; it also produces distortion that is specific to the mechanisms being used. Intentional forgetting, as demonstrated by Festini and Reuter-Lorenz [42], can eliminate proactive interference during working memory tasks, thus demonstrating that cognitive control can be maintained during a cognitive load. These effects that arise from cognitive load are magnified in aging and clinical populations. For instance, Sacripante et al. [43] reported that individuals with mild cognitive impairment (MCI) resulting from Alzheimer’s disease had poorer initial encoding and showed a greater degree of early forgetting over relatively short retention intervals, implying a reduced ability to stabilize encoded memories. Gagnon and Belleville [44] reported that increased intrusion errors in the context of Alzheimer’s disease resulted from a relative lack of inhibitory control and demonstrated a greater susceptibility to being interfered with compared to control groups (Table 1).

### 3.7. Adaptive Forgetting

Adaptive forgetting is the goal-oriented, context-dependent decrease in memory accessibility that improves performance by reducing interference, conserving resources, and increasing flexibility. There is considerable empirical evidence that supports the view of active suppression playing a role in forgetting, to some extent, but not yet any direct comparison of this theory to other alternative models (decay, interference, or failure-to-retrieve models), which raises the issue regarding how much of the adaptive process of forgetting is based on active or passive mechanisms.

#### 3.7.1. Competition and Interference Resolution

Retrieval competition refers to the process whereby the retrieval of a target item enhances its accessibility and reduces the recall of other competing items. Interference and the prioritization of goal-relevant information were evident due to the limited capacity of working memory [4]. The contextual updating process was shown to decrease dependence on irrelevant information in an ever-changing context [16]. There have also been cases of retrieval-induced forgetting for emotionally laden material [45], and there is evidence from brain imaging studies showing cortical inhibition of competing memory traces [2].

#### 3.7.2. Implicit Adaptive Decay in Working Memory

Adaptive decay has been proposed as a mechanism that down-regulates representations of outdated items in working memory. Longer response time was observed with greater numbers of intervening operations, a pattern consistent with intentional suppression of inactive information [40]. It was observed that demonstrated that intentional forgetting similarly reduces proactive interference, suggesting that forgetting can be strategically deployed within working memory [1,42]. Episodic details were found to be forgotten more rapidly than gist, due to resource optimization [46].

#### 3.7.3. Evidence from Computational Models and Optimization

Models of reinforcement learning demonstrate that forgetting decayed associations speeds up learning in variable environments [22,47,48,49,50]. Forgetting, tuned to volatility (i.e., adapting to environmental change), enhances learning. Adaptive forgetting has four roles: decreasing interference [4,40], increasing flexibility [16,22,47,48,49,50], maximizing working memory capacity [42], and promoting system stability [1]. Intermediate levels of neural activity during suppression have also been found to predict greater forgetting, suggesting the possibility of an optimal inhibitory zone [51].

### 3.8. Encoding Vulnerability and Accelerated Early Forgetting in MCI and Alzheimer’s Disease

Sacripante et al. [43] showed that AD MCI patients have weaker encoding and faster initial forgetting over short retention intervals than older adults. Critically, accelerated forgetting was not caused by accelerated long-term forgetting but by poor encoding and lack of post-learning consolidation. Wearn et al. [13] showed that accelerated long-term forgetting (ALF) predicts cognitive decline over 12 months and is associated with hippocampal volumetry, indicating ALF reflects early progressive neurodegenerative processes. Gagnon and Belleville [44] observed that AD patients show increased intrusion errors, decreased semantic clustering, and increased forgetting after longer delays. Age-related effects on intentional forgetting are shown by reduced prefrontal activity [52], reflecting decreased inhibitory ability.

### 3.9. Reduced Hippocampal Encoding and Memory Fragmentation in Post-Traumatic Stress Disorder (PTSD)

It has been demonstrated that trauma-exposed people with PTSD have decreased hippocampal and amygdala activity during the encoding of emotional stimuli, and higher rates of false alarms for new images. Lower left hippocampal activation was associated with symptom severity, suggesting that dysfunction of the medial temporal lobe (MTL) underlies fragmented, labile memories in PTSD [17]. Dolcos et al. [53] found that dissociation within the MTL, amygdala aids encoding of emotional items, the entorhinal cortex supports item identity, and the hippocampus is responsible for binding items to context. This accounts for the finding that PTSD patients have intact emotional item memory but impaired contextual memory, which is a pathological type of forgetting. Several studies [48,54] report overgeneral memory, where people remember categories of events. This stems from impaired inhibition and the inability to suppress intrusions, resulting in episodic details not being retrieved.

### 3.10. Lack of Specificity, Negativity Bias, and Network Dysfunction in Major Depressive Disorder

**Figure 6:**
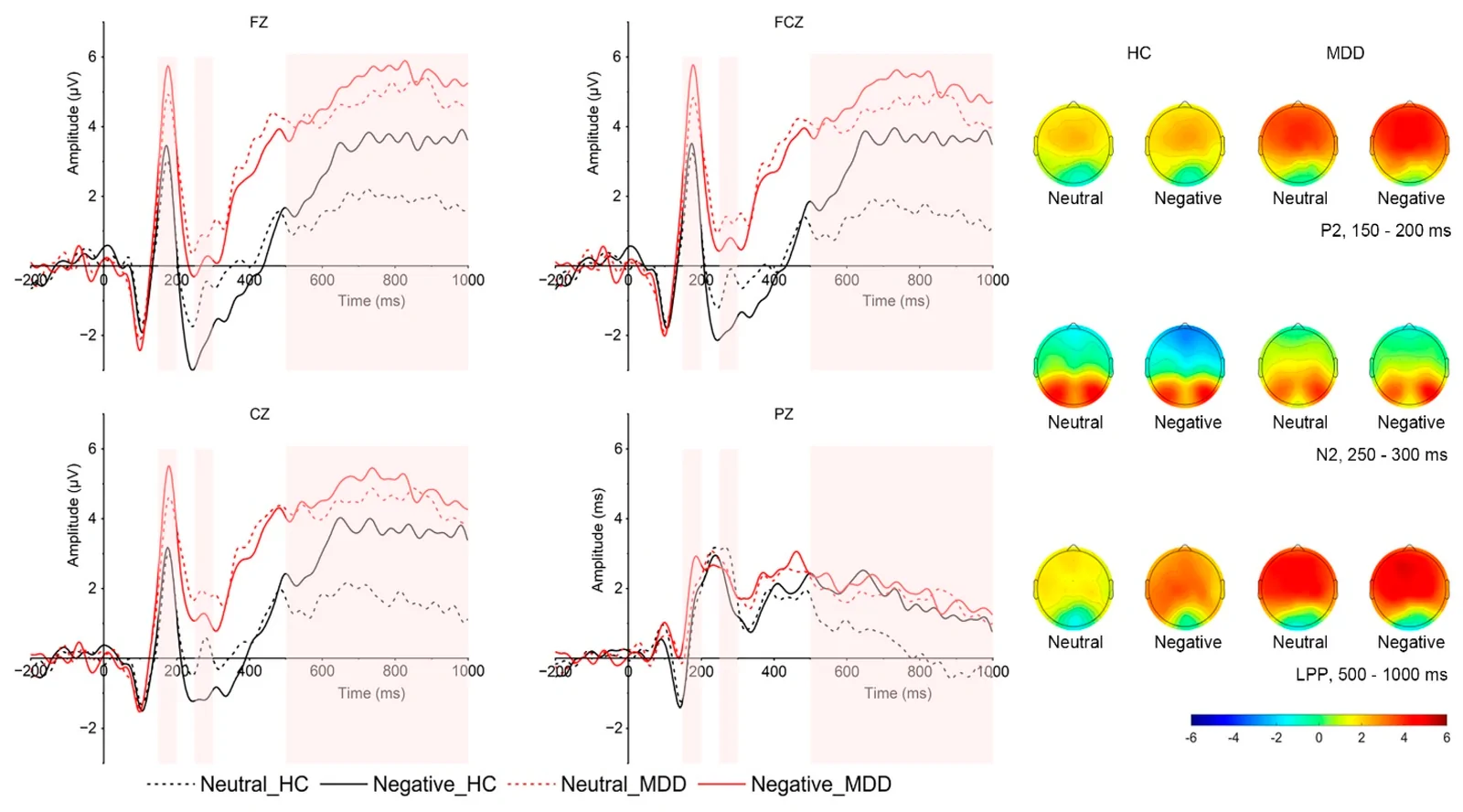
Grand-averaged ERPs in frontal (FZ), frontocentral (FCZ), central (CZ), and parietal (PZ) locations and topographical distribution of grand-averaged image-evoked P2, N2, and LPP of both MDD and HC groups. The image-evoked P2 time window is 150–200 ms; the image-evoked N2 time window is 250–300 ms; the image-evoked LPP time window is 500–1000 ms. LPP, late positive potential; MDD group, major depressive disorder group; HC, healthy control group; ERP, event-related potentials [10]. Adapted under the Creative Commons Attribution License (CC BY 4.0).

**Figure 7:**
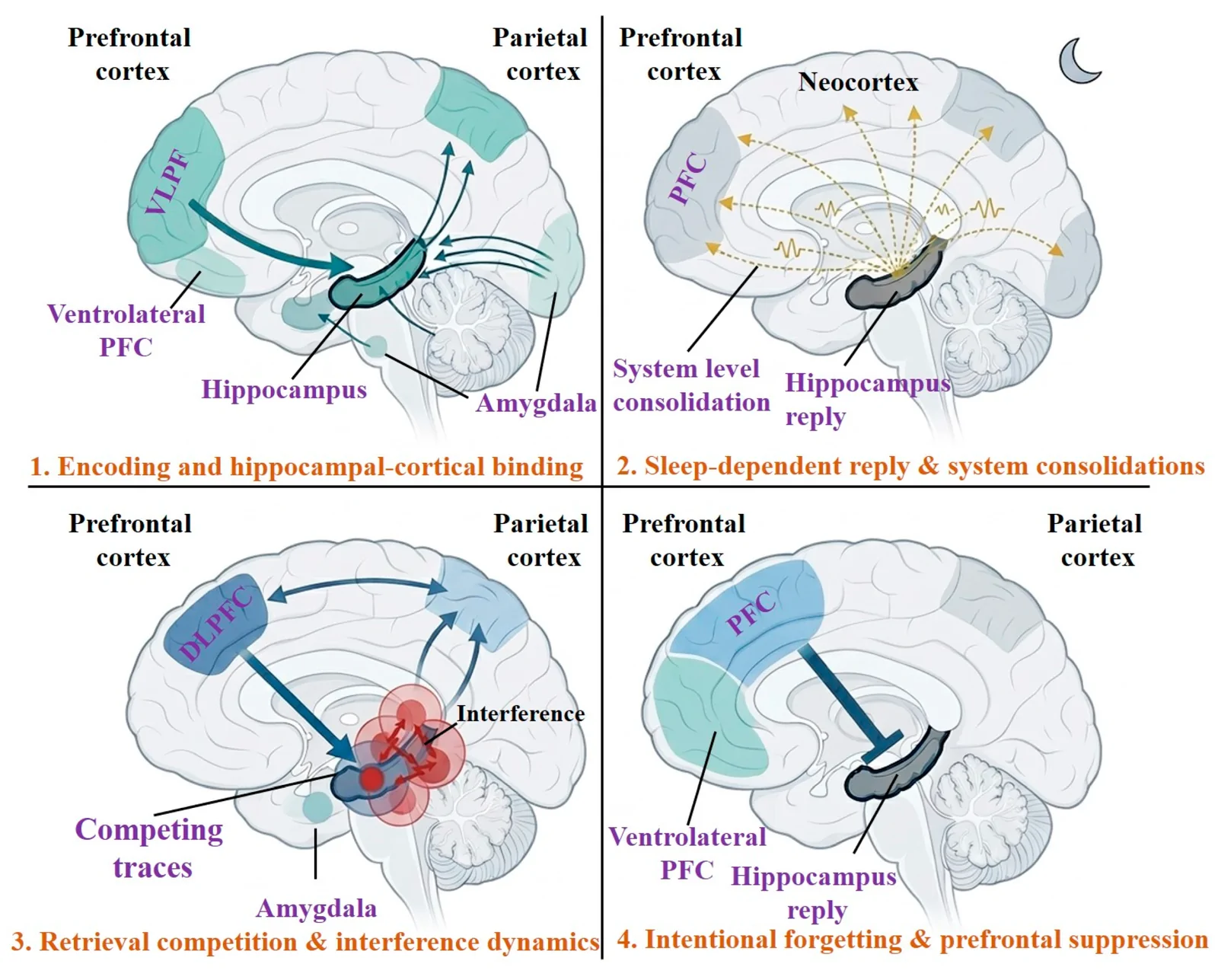
The original schematic created by the authors demonstrated neural systems of memory: encoding, consolidation, retrieval, and forgetting. The figure illustrates the major neural systems underlying memory processes and the mechanisms of forgetting. (**1**) Encoding and hippocampal–cortical binding are supported by ventrolateral prefrontal cortex [VLPFC] modulation of hippocampal activity, enabling the formation of new memory traces. (**2**) Sleep-dependent replay and systems consolidation involve hippocampal reactivation that drives long-term neocortical integration. (**3**) Retrieval competition and interference dynamics arise when overlapping memory traces compete in the hippocampus, regulated by the dorsolateral prefrontal cortex [DLPFC]. (**4**) Intentional forgetting occurs through top-down prefrontal suppression of hippocampal activity, reducing accessibility of unwanted or irrelevant memories. Color legend: Blue: Encoding/color formation; Yellow: Sleep dynamics; Red: Interference/failed retrieval; Gray: Suppression/inhibition.

MDD patients have difficulty retrieving autobiographical memories with high specificity; they fail to recall episodic details and instead retrieve or access general or semantic information [55,56]. This is associated with weakened executive control, especially in blocking out irrelevant negative information, which undermines episodic memory. Another feature of MDD is greater encoding and retrieval of negative items, and impaired recall of neutral or positive items [53]. This results in selective pathological forgetting, where emotion biases, rather than decay, contribute to pathological memory. Imaging research has reported decreased volume of the hippocampus and changed prefrontal cortex activation in MDD, leading to encoding and retrieval monitoring deficits. This impairs memory vividness, detail, and retrieval efficiency [57] (Figure 6 and Figure 7).

## 4. Discussion

In healthy adults, intentional forgetting was consistently demonstrated using directed forgetting and Think/No-Think paradigms. Behavioral suppression of recall was associated with reproducible neural signatures of executive inhibition, including increased frontoparietal control activity and reduced hippocampal engagement during suppression attempts. These patterns are consistent with the possibility that forgetting may be deliberately initiated and neurally implemented and are difficult to reconcile with purely passive decay models, though decay and interference theories have not been conclusively rejected but continue to be debated [7,26,58,59,60,61].

Inhibitory Control Model is supported by the consistent finding of a reduction in prefrontal–hippocampal network activation during intentional suppression in healthy adults [7,8] and the inability of such a suppression mechanism in PTSD and MDD patients [10,17]. The second theory, interference theory, is backed by the retrieval competition results in which recall of the target memories caused a decrease in accessibility of the competing memories [2,3]. Finally, the third model, the Executive Resource Model, is supported by studies showing that the cognitive load and psychological stress had negative effects on working and prospective memory, and were correlated to the level of cortisol [4,12]. These three theories complement each other in explaining the processes of forgetting in humans [24,25,42]. Electrophysiological evidence further suggested that intentional forgetting and suppression are associated with distinct temporal dynamics of cognitive control, including enhanced frontal N2 components, reduced parietal P3 and late positive complex amplitudes, and increased alpha and theta oscillatory power during suppression trials. These findings are consistent with the view that forgetting may involve active allocation of attentional and inhibitory resources at encoding and retrieval stages, though the precise causal role of these markers remains to be established [7,59].

Another dimension of relevance to intentional forgetting was age-related changes in inhibitory control. Rizio and Dennis have found age-related differences in the brain regions associated with memory and forgetting success, implying that the neuronal mechanisms underlying memory control are subject to change with age regardless of any pathology [52]. Interplay between age and emotion factors is also observed during memory suppression since emotion content was found to impact the results of the think/no-think task differently depending on age [30]. Thus, one could suggest that age-related deficits in inhibitory control might be seen as normative antecedents to, or risk factors for, more severe suppression impairments characteristic of the pathological groups like MCI, AD, PTSD, and MDD.

In clinical and aging populations, forgetting was not uniformly accelerated but instead appeared to reflect stage-specific impairments, primarily at encoding and executive control levels. In mild cognitive impairment and Alzheimer’s disease, poorer immediate recall and increased sensitivity to retention intervals are consistent with encoding and working memory limitations rather than indiscriminate long-term trace loss [13,43,44]. Notably, studies employing methodologically rigorous designs found that, when encoding strength was equated, long-term forgetting rates in Alzheimer’s disease were comparable to those of healthy controls, further challenging the notion of generalized accelerated forgetting [14]. The present observation contrast to previous findings of accelerated long-term forgetting in AD patients [13] because the difference between them lies in the inconsistency between the control of encoding strength in both designs, as failure to control encoding could easily account for a deficit at the encoding stage being falsely attributed to accelerated forgetting. Stress-related psychiatric conditions provided additional, though largely correlational, support for an active-control framework. Patients with posttraumatic stress disorder and major depressive disorder showed selective failures in suppressing unwanted or negative memories, accompanied by aberrant hippocampal, amygdalar, and prefrontal activity during encoding and control attempts. These findings are consistent with the hypothesis that pathological forgetting in these populations may relate to disrupted inhibitory control and stress-sensitive memory modulation, rather than global memory erosion, although directionality cannot be firmly established from the available human data [4,17,26,49,62]. Sekeres et al. (2016) demonstrated that even in healthy adults, forgetting rates are not uniform across all features of an episode; peripheral, contextual details were forgotten more rapidly than central, gist-like information, suggesting that differential forgetting of detail types is a fundamental property of episodic memory [63]. This finding is consistent with the idea that there are both active-suppression mechanisms and interference-based mechanisms. But it does not conclusively determine which explanation is more compelling. Lee et al. [64] observed that using an item-method directed forgetting task, intentional forgetting reduced perceptual interference for to-be-forgotten items at a perceptual rather than semantic level, suggesting that the active allocation of processing resources away from unwanted representations may contribute to forgetting even in non-clinical populations. Consequently, Noreen et al. [65] have demonstrated that directly and indirectly suppressing autobiographical memories involve different areas of the brain, lending more support to the notion that forgetting is accomplished via specific control processes rather than via one general mechanism.

Forgetting can also occur via processes largely unaffected by executive processes. Systematic consolidation involves reorganization of memory traces through the hippocampal cortical network system, and systematic consolidation impairment could be another cause of forgetting [22]. The retrieval process may result in destabilization of the consolidated memory, which is then restabilized by reconsolidation; any disruption to this process is thought to present another forgetting mechanism that takes place associated with retrieval [66]. Synaptic downscaling through sleep renormalizes the synaptic strength and weakens synapses of less relevance while prioritizing synapses engaged in strong hippocampal activation during encoding [21,67].

These mechanisms provide a theoretical basis different from the inhibitory control mechanisms outlined previously, and the comparative explanatory success of executive and systems consolidation theories continues to be disputed. The following examples of non-executive forgetting mechanisms show that not all forms of forgetting depend on executive function. Future studies should determine the extent to which the forgetting that occurs among the above populations results from these forgetting mechanisms.

Biomarker validation approaches require proofs of analytical validity, clinical validity, and clinical utility [68]. In the case of Alzheimer’s disease and mild cognitive impairment, behavioral measures of forgetting accelerated forgetting rates [13,43] and impairments in working memory; the measurements are done via neuropsychological tests, such as ADAS-Cog, MMSE, and MoCA, which already have established clinical norms, which is why these tools are practically useful [69]. In contrast, neuroimaging results obtained on PTSD patients, demonstrating impaired functioning of the hippocampus/amygdala [17,49], and patients suffering from major depression, with changes in ERP [10] and cortisol [62] levels, confirm clinical validity but lack clinical utility; they do not have standardized clinical tests associated with them and do not have standardized cutoff amounts [68]. Studies of direct brain stimulation (TMS/tDCS) provide evidence that prefrontal–hippocampal circuits are involved in the suppression of memories [24,25], suggesting potential targets for intervention, but clinical implementation of results is still in progress. Therefore, behavioral measures are more useful now for real clinical application than neuroimaging biomarkers, which require further testing [68]. These insights into the mechanisms provide possible directions for potential applications, but the link between an intervention and its effect on outcome has not been demonstrated yet. Considering the causal role of prefrontal–hippocampal connectivity in retrieving-induced forgetting [24,25], neurostimulation methods such as TMS and tDCS might be one possible method of memory manipulation suppression in cases when this process is dysfunctional because of inhibited control in PTSD. Indeed, as there is a relation between the capacity for memory suppression and resilience from trauma [26] and it is possible to induce suppression experimentally [27], this ability can be modified and not hardwired. With respect to MCI and early Alzheimer’s disease, as the process of forgetting in these disorders is associated with failures during encoding and consolidation rather than acceleration of decay processes [13,14,43], interventions focusing on these processes could prove more beneficial than those that would focus on retrieving. There are several unanswered questions, as the issue of accelerated forgetting in AD/MCI as a result of encoding failure, consolidation problems, or accelerated decay remains unclear because of contradicting results between studies that equated encoding strength and those that did not [13,14,43]. It is also not clear if there is a cause-and-effect relationship between the impaired function of inhibitory control and pathological forgetting in PTSD/MDD patients [4,17,26,49,62].

## 5. Conclusions

Executive and emotional-regulatory system compromise produces maladaptive forgetting characterized by reduced specificity, increased interference, and impaired inhibition. Stress shifts memory toward gist-based representations, reducing contextual detail and altering the balance between generalization and specificity. In PTSD and MDD, failure to suppress unwanted or negative memories correlates with dysregulated hippocampal, amygdalar, and prefrontal activity, reflecting inhibitory control deficits that produce asymmetric forgetting: intrusive emotional memories persist pathologically while contextual details are selectively lost. Collectively, findings across healthy, aging, and clinical populations support the hypothesis that forgetting is a dynamic, actively regulated cognitive process shaped by executive control, hippocampal temporal dynamics, emotional salience, and neural vulnerability. Disruptions to any component yield distinct pathological forgetting profiles, underscoring the need for mechanistic frameworks in accurate interpretation. From the clinical perspective, these mechanistic differences indicate that different treatments should be developed based on the disrupted process: encoding and consolidation of memories in MCI and control processes of inhibition in PTSD and MDD. In order to realize this goal, future research is needed to develop biomarkers with clinical significance.

### 5.1. Strength of the Study

The current review has several advantages, including behavioral paradigms, neuroimaging techniques, and electrophysiological methods that were utilized, thereby ensuring triangulation at different levels of analysis, as evidenced by convergent findings in fMRI, EEG, intracranial EEG, PET, and structured cognitive tasks, and thus creating a solid multi-modal basis for studying forgetting mechanisms. Clinical and older adults have used performance-matched learning paradigms, removing the differences in the level of initial acquisition as an extraneous variable and thus improving the conclusions about the nature of forgetting patterns, especially in MCI and AD. Paradigms assessing intentional suppression, interference resolution, emotion processing, and stress retrieval provided an opportunity to investigate forgetting at different cognitive-affective levels. Both behavioral and neural measures were used, enabling cross-validation, as electrophysiological markers (frontal N2, theta/alpha activity) were consistently associated with behavioral success in suppression, thereby confirming causality in forgetting mechanisms.

### 5.2. Limitations of the Study

There were a few limitations, such as inaccurate recall and higher false alarms or decay rates, and limited quantifiable comparison between them. Although task paradigms used different procedures, including directed forgetting, think-or-no-think, and emotional memory tasks, the processes involved in each were overlapping yet not identical, making it difficult to determine whether an effect was attributable to attention disengagement, inhibition, or working memory demands. In addition, neuroimaging sample sizes were relatively small, and clinical samples were heterogeneous in terms of disease stages, history of trauma, and comorbidity, specifically among MCI and PTSD individuals, which made it difficult to determine the link between inhibitory control and forgetting behaviors. In this context, most of the findings, especially those relating to PTSD and MDD, have been correlational, while causal evidence has only been found in a few studies conducted in healthy participants, and so the causality between inhibitory control and pathological forgetting remains to be determined. Lastly, none of the reviewed literature used any intervention to test the impact of the clinical applications suggested, and therefore, despite being inferential, they require clinical trials to confirm their validity.

## Figures and Tables

**Table 1:** Summary of design, population, methodology, and principal findings of the selected studies.

References	Study Design	Population	Methodology	Principal Findings
Oberauer & Greve [1]	Experimental	Adults	Cognitive task performance	Intentional forgetting operates across working and long-term memory systems
Wimber et al. [2]	Experimental/fMRI	Healthy adults	Neuroimaging (fMRI)	Retrieval-induced forgetting involves cortical pattern suppression of competitors
García-Rueda et al. [3]	Experimental	Healthy adults	Visual memory task with controlled interference	Both decay and interference contribute to visual memory forgetting
De Jong et al. [4]	Experimental	Healthy adults	Working memory task	Forgetting rate in working memory adapts to task demands
Parker et al. [5]	Experimental	Adults	Item-method directed forgetting task	Survival processing can modulate directed forgetting effects
Chalkia et al. [6]	Experimental	Healthy adults	Behavioral and physiological measures	Directed forgetting can suppress emotional associative memories
Gao et al. [7]	Experimental/EEG	Healthy adults	ERP recording	Frontal cortex engagement during intentional forgetting
Pan & Li [8]	Experimental/EEG	Healthy adults	Behavioral tasks and EEG	Different forgetting strategies show distinct neural patterns
Topçu et al. [9]	Neuroimaging/iEEG	Surgical patients	Intracranial recordings	Left anterior prefrontal cortex critical for verbal memory encoding
Chen et al. [10]	Clinical/ERP	Depressed patients + controls	ERP recording	MDD shows altered neural response during emotional directed forgetting
Santangelo et al. [11]	Experimental/fMRI	Superior memory individuals + controls	fMRI during directed forgetting task	HSAM shows different neural signatures during intentional forgetting
Matos et al. [12]	Experimental	Adults	Prospective memory task with load manipulation	Cognitive load increases prospective memory commission errors
Cantarella et al. [33]	Experimental/fMRI	Healthy adults	fMRI during prospective memory task	Cognitive load and task focality interact in prospective memory
Wearn et al. [13]	Longitudinal	Healthy older adults	Memory testing + cognitive assessment	Accelerated forgetting predicts cognitive decline risk
Stamate et al. [14]	Clinical	Alzheimer’s patients + controls	Recall and recognition tasks	Forgetting patterns differ in Alzheimer’s disease
Lohnas et al. [15]	Neuroimaging/intracranial	Surgical patients	Intracranial recordings during encoding	Neural fatigue during encoding predicts memory performance
Cortese et al. [16]	Experimental/fMRI	Healthy adults	Fear conditioning task with fMRI	Neural circuits arbitrate between different memory types over time
Hayes et al. [17]	Clinical/fMRI	Combat veterans with PTSD	fMRI during memory task	Reduced medial temporal activity associated with memory distortions in PTSD
Kensinger & Schacter [18]	Experimental/fMRI	Healthy adults	fMRI during encoding	Amygdala enhances item memory but not source memory for emotional stimuli
Kuhl et al. [19]	Neuroimaging/fMRI	Healthy adults	fMRI pattern analysis during encoding	Encoding patterns predict later memory success
Basso Moro et al. [20]	Neuroimaging/fNIRS	Healthy adults	fNIRS during verbal memory task	Prefrontal cortex shows differential activation for encoding vs. retrieval
Rauchs et al. [21]	Experimental/neuroimaging	Healthy adults	Memory testing with sleep manipulation	Sleep selectively strengthens memories based on learning-phase hippocampal activity
St Jacques et al. [23]	Neuroimaging/fMRI	Healthy adults	fMRI during retrieval task	Distributed neural networks support memory retrieval
Penolazzi et al. [24]	Experimental/TMS	Healthy adults	TMS with behavioral measures	Causal evidence for inhibitory control in memory retrieval
Khan et al. [25]	Experimental/tDCS	Healthy adults	Stimulation with electrophysiology	mPFC stimulation modulates retrieval-induced forgetting
Mary et al. [26]	Clinical	Trauma survivors	Memory and psychological assessment	Memory suppression capacity linked to trauma resilience
Hulbert et al. [27]	Experimental	Healthy adults	Behavioral inhibition paradigm	Systematic suppression can induce targeted forgetting
Schönfeld et al. [28]	Experimental	Healthy adults	Stress manipulation with pharmacological measures	Arousal and glucocorticoids have distinct effects on memory
Marchewka et al. [29]	Experimental	Healthy adults	Directed forgetting task with emotional manipulation	Basic emotions modulate directed forgetting effectiveness
Murray et al. [30]	Experimental	Young and older adults	Think/no-think directed forgetting task	Age and emotion interact in intentional forgetting
Dreifus et al. [31]	Experimental	Healthy adults	Stress induction with behavioral task	Stress does not eliminate retrieval-induced forgetting
Zoladz et al. [32]	Experimental	Healthy adults	Stress with hormonal measurement	Sex and stress response predict stress effects on memory
Khan et al. [34]	Experimental	Healthy adults	Prospective memory tasks varying load	Cognitive load differentially affects event vs. time-based PM
Meier & Zimmermann [35]	Experimental	Healthy adults	Multiple load manipulations in PM task	Multiple load types independently affect prospective memory
Rummel et al. [36]	Experimental	Healthy adults	Prospective memory with action coordination	Action coordination demands affect prospective memory realization
West et al. [37]	Experimental/fMRI	Healthy adults	fMRI during prospective memory with load	Working memory load modulates prospective memory brain activation
Oei et al. [38]	Experimental	Healthy adults	Stress induction with memory tasks	Stress impairs high-load working memory via cortisol
Lupien et al. [39]	Experimental	Healthy adults	Cortisol administration with memory tasks	Working memory more sensitive to acute corticosteroid effects
Altmann & Gray [40]	Experimental	Healthy adults	Memory tasks isolating decay vs. interference	Decay and interference are functionally related processes
Sun et al. [41]	Experimental	Healthy adults	Memory task manipulating interference types	Different interference types affect memory aspects differently
Festini & Reuter-Lorenz [42]	Experimental	Healthy adults	Directed forgetting with interference measures	Directed forgetting reduces working memory interference
Sacripante et al. [43]	Clinical	MCI patients + controls	Prose recall testing	Accelerated forgetting characterizes MCI
Gagnon & Belleville [44]	Clinical	MCI/AD patients + controls	Working memory span tasks	Forgetting contributes to WM deficits in MCI/AD
Reeck & LaBar [45]	Experimental/fMRI	Healthy adults	fMRI during retrieval-induced forgetting with emotion	Retrieval-induced forgetting applies to emotional memories
Sadeh et al. [46]	Experimental	Healthy adults and amnesic patients	Recognition and recall tasks	Different forgetting patterns indicate distinct memory representations
Aslan et al. [47]	Developmental	Young children	Directed forgetting task	Children show production deficiency in directed forgetting
Zellner & Bäuml [48]	Developmental	Children	Retrieval inhibition paradigm	Retrieval inhibition intact in children
Zhang et al. [22]	Neuroimaging/intracranial	Surgical patients	Intracranial recordings during sleep	Electrophysiological signatures of memory consolidation
Admon et al. [49]	Neuroimaging/fMRI	Healthy adults with stress reactivity measure	fMRI + stress task	Individual differences in amygdala and hippocampus predict stress vulnerability
Schwabe et al. [50]	Experimental	Healthy adults	Stress + memory task + pharmacological manipulation	Beta-adrenergic blockade prevents stress effects on memory
Detre et al. [51]	Experimental	Healthy adults	Think/no-think task with activation measure	Moderate activation during suppression induces forgetting
Rizio & Dennis [52]	Experimental/fMRI	Young and older adults	fMRI during think/no-think task	Age-related differences in neural control of memory
Dolcos et al. [53]	Experimental/fMRI	Healthy adults	fMRI during emotional encoding	Amygdala-MTL interaction predicts emotional memory
Kuhlmann & Wolf [54]	Experimental	Healthy young men	Memory task with arousal/cortisol measurement	Arousal and cortisol interact in consolidation
Eldridge et al. [55]	Neuroimaging/fMRI	Healthy adults	fMRI during encoding and retrieval	Hippocampus shows distinct encoding vs. retrieval signatures
Kuhlmann et al. [56]	Experimental	Healthy young women	Cortisol administration + memory task	Cortisol impairs retrieval of negative memory
Richardson et al. [57]	Experimental/fMRI	Healthy adults	fMRI during emotional encoding	Amygdala-hippocampus interaction encodes emotional memories
Gamboa et al. [58]	Experimental/fMRI	Healthy adults	fMRI during auditory directed forgetting	Auditory domain shows similar directed forgetting mechanisms
Depue et al. [59]	Experimental/EEG	Healthy adults	ERP and oscillations during suppression	Theta and beta oscillations during memory suppression
Rizio & Dennis [60]	Experimental/fMRI	Healthy adults	fMRI during think/no-think task	Prefrontal-parietal networks control intentional forgetting
Bastin et al. [61]	Experimental/fMRI	Healthy adults	fMRI during directed forgetting	Right prefrontal cortex supports memory suppression
Grossman et al. [62]	Clinical/experimental	PTSD patients + controls	Hydrocortisone administration + cognitive testing	Glucocorticoid effects on cognition differ in PTSD
Sekeres et al. [63]	Experimental	Healthy adults	Memory testing for different detail types	Different memory details are forgotten at different rates
Lee et al. [64]	Experimental	Healthy adults	Directed forgetting with Stroop-like interference	Directed forgetting reduces automatic interference effects
Noreen et al. [65]	Experimental/fMRI	Healthy adults	fMRI during autobiographical memory suppression	Direct and indirect suppression engage distinct neural regions

## Data Availability

No new data were created or analyzed in this study. Data sharing is not applicable to this article.

## Supplementary Materials

The following supporting information can be downloaded at: https://www.mdpi.com/article/10.3390/medicina62081550/s1, File S1. PRISMA 2020 Checklist [70].

## Abbreviation

The following abbreviations are used in this manuscript:

AD	Alzheimer’s Disease
ADAS-Cog	Alzheimer’s Disease Assessment Scale-Cognitive subscale
ALF	Accelerated Long-Term Forgetting
CC BY	Creative Commons Attribution
DLPFC	Dorsolateral Prefrontal Cortex
EEG	Electroencephalography
ERP	Event-Related Potentials
fMRI	Functional Magnetic Resonance Imaging
fNIRS	Functional Near-Infrared Spectroscopy
HC	Healthy Control
HFA	High-Frequency Activity
HSAM	Highly Superior Autobiographical Memory
Hz	Hertz
iEEG	Intracranial EEG
LPP	Late Positive Potential
MCI	Mild Cognitive Impairment
MDD	Major Depressive Disorder
MMSE	Mini-Mental State Examination
MoCA	Montreal Cognitive Assessment
mPFC	Medial Prefrontal Cortex
ms	Milliseconds
MTL	Medial Temporal Lobe
nREM	Non-REM Sleep
NOS	Newcastle-Ottawa Scale
OTL	Ongoing Task Load
PET	Positron Emission Tomography
PL	Prospective Load
PRISMA	Preferred Reporting Items for Systematic Reviews and Meta-Analyses
PTSD	Post-Traumatic Stress Disorder
REM	Rapid Eye Movement Sleep
RL	Retrospective Load
tDCS	Transcranial Direct Current Stimulation
TMS	Transcranial Magnetic Stimulation
VLPFC	Ventrolateral Prefrontal Cortex
